# Cybathlon experiences of the Graz BCI racing team Mirage91 in the brain-computer interface discipline

**DOI:** 10.1186/s12984-017-0344-9

**Published:** 2017-12-28

**Authors:** Karina Statthaler, Andreas Schwarz, David Steyrl, Reinmar Kobler, Maria Katharina Höller, Julia Brandstetter, Lea Hehenberger, Marvin Bigga, Gernot Müller-Putz

**Affiliations:** 1 Institute of Neural Engineering, Laboratory of Brain-Computer Interfaces, Graz University of Technology, 8010 Graz, Austria; 2 Graz BCI Racing Team MIRAGE91, Graz University of Technology, Graz, Austria

**Keywords:** Brain-computer Interface (BCI), Electroencephalogram (EEG), Stroke, CYBATHLON, Mental imagery

## Abstract

**Background:**

In this work, we share our experiences made at the world-wide first CYBATHLON, an event organized by the Eidgenössische Technische Hochschule Zürich (ETH Zürich), which took place in Zurich in October 2016. It is a championship for severely motor impaired people using assistive prototype devices to compete against each other. Our team, the Graz BCI Racing Team MIRAGE91 from Graz University of Technology, participated in the discipline “Brain-Computer Interface Race”. A brain-computer interface (BCI) is a device facilitating control of applications via the user’s thoughts. Prominent applications include assistive technology such as wheelchairs, neuroprostheses or communication devices. In the CYBATHLON BCI Race, pilots compete in a BCI-controlled computer game.

**Methods:**

We report on setting up our team, the BCI customization to our pilot including long term training and the final BCI system. Furthermore, we describe CYBATHLON participation and analyze our CYBATHLON result.

**Results:**

We found that our pilot was compliant over the whole time and that we could significantly reduce the average runtime between start and finish from initially 178 s to 143 s. After the release of the final championship specifications with shorter track length, the average runtime converged to 120 s. We successfully participated in the qualification race at CYBATHLON 2016, but performed notably worse than during training, with a runtime of 196 s.

**Discussion:**

We speculate that shifts in the features, due to the nonstationarities in the electroencephalogram (EEG), but also arousal are possible reasons for the unexpected result. Potential counteracting measures are discussed.

**Conclusions:**

The CYBATHLON 2016 was a great opportunity for our student team. We consolidated our theoretical knowledge and turned it into practice, allowing our pilot to play a computer game. However, further research is required to make BCI technology invariant to non-task related changes of the EEG.

## Background

In October 2016, a novel event called CYBATHLON, organized by the Eidgenössische Technische Hochschule Zürich (ETH Zürich), took place in Zurich, Switzerland, for the first time [1]. The vision of this event is to provide a platform for pilots with severe motor impairments to compete against each other with the support of technical assistive systems and to drive forward their development [2].

The competition is composed of six different disciplines, according to the respective type of assistive system the pilots are using. The disciplines are: Functional Electrical Stimulation Bike Race, Powered Arm Prosthesis Race, Powered Leg Prosthesis Race, Powered Exoskeleton Race, Powered Wheelchair Race, and Brain-Computer Interface (BCI) Race. The races are designed to test the ability of pilots to navigate through a series of everyday tasks within minimal time. Details can be found on the CYBATHLON homepage [1].

Besides the pilot, the supporting team of caregivers and engineers is a key factor in a successful participation in any of the disciplines. The competition between pilots is thus, by extension, a competition between teams. The Graz BCI Lab formed a team named “MIRAGE91” to compete in the BCI Race discipline [3, 4].

A BCI is a device that enables users to interact with their environment by intentionally modulating their brain activity [5]. The non-invasive Graz-BCI focuses on the changes of oscillatory components in electroencephalography (EEG) signals due to different mental tasks, like motor imagery or mental arithmetic [6, 7]. It translates the changes into computer commands to control an application. Potential BCI-related applications include spelling devices [8] painting [9] or even music composing [10]. Furthermore, control scenarios like upper arm motor neuroprosthesis [11–14] or wheelchair control [15, 16] are investigated. In the case of the BCI Race, the application is a computer game. The game “BrainRunners” was specifically developed for the CYBATHLON competition and provided to the teams in advance to enable them to efficiently prepare for the race. The pilot controls an avatar in a race against up to three competitors. The avatar continuously moves forward along a straight race track. The race track itself consists of a pseudorandom sequence of pads, i.e. three different action pads and one rest pad. The avatar receives a speed boost on action pads if the pilot sends the right command with regard to the field, but is slowed down whenever a wrong command is triggered. On rest pads, there is no correct command, but the avatar is slowed down with any command. Therefore, in the optimal case, the pilot is able to control four different commands reliably (no command and 3 action commands) [1].

This paper aims at sharing the experiences of the Graz BCI Racing Team MIRAGE91 gathered at the CYBATHLON 2016. We describe the preparations, starting from how we formed the team and found our pilot, to our multi-stage training procedure to individualize and adapt the BCI technology to our pilot, and the final BCI technology setup in chapter 2. We report on the practical knowledge we have gained at the event itself in chapter 3, and finally, we discuss organizational challenges, the positive public awareness, future plans and close with lessons learned in chapter 4.

## Preparations

### MIRAGE91 - the Graz BCI racing team - familiarizing students with BCI research

Since the BCI field [17, 18], is very interdisciplinary, it requires knowledge and expertise from many areas such as neurophysiology, anatomy, psychology, neuroscience, computer science, biomedical engineering, electronics, software engineering, machine learning, statistics et cetera. Bringing students into the field usually involves disproportional effort, not only for the educator but also for students themselves. One of our strategies to introduce students into BCI early on is to offer classes at master level in several study programs. Additionally, the BCI Lab of the Graz University of Technology has founded the Graz BCI Racing Team.

During courses in our study programs Information and Computer Engineering and Biomedical Engineering, we announced the idea of establishing a team to participate in the BCI Race and asked for interested students. In October 2014, we started with first informative meetings; we developed the idea, explained the CYBATHLON and highlighted several tasks in such a team: BCI development, creation of paradigms for training, analysis of the BCI Race game, search for potential pilots, organization of pilot training, maintenance of a website, public relations, sponsoring, and team outfit. In this way, we were able to shape a loose group of students into the Graz BCI Racing Team, named MIRAGE91 (Motor Imagery Racing Graz established 1991, the year when BCI research started in Graz). Our BCI Racing Team consists of PhD, Master, and Bachelor level students of the study programs Information and Computer Engineering, Biomedical Engineering, Computer Science and Mathematics. The team was announced officially by the university and has its own website [4].

As one of the first activities, we participated in the CYBATHLON rehearsal in July 2015, where we were able to familiarize ourselves with the competition handling, our BCI, and available infrastructure. This was of special importance, since we needed to know how to organize our participation in the actual championship in October 2016 with a severely handicapped pilot.

**Fig. 1:**
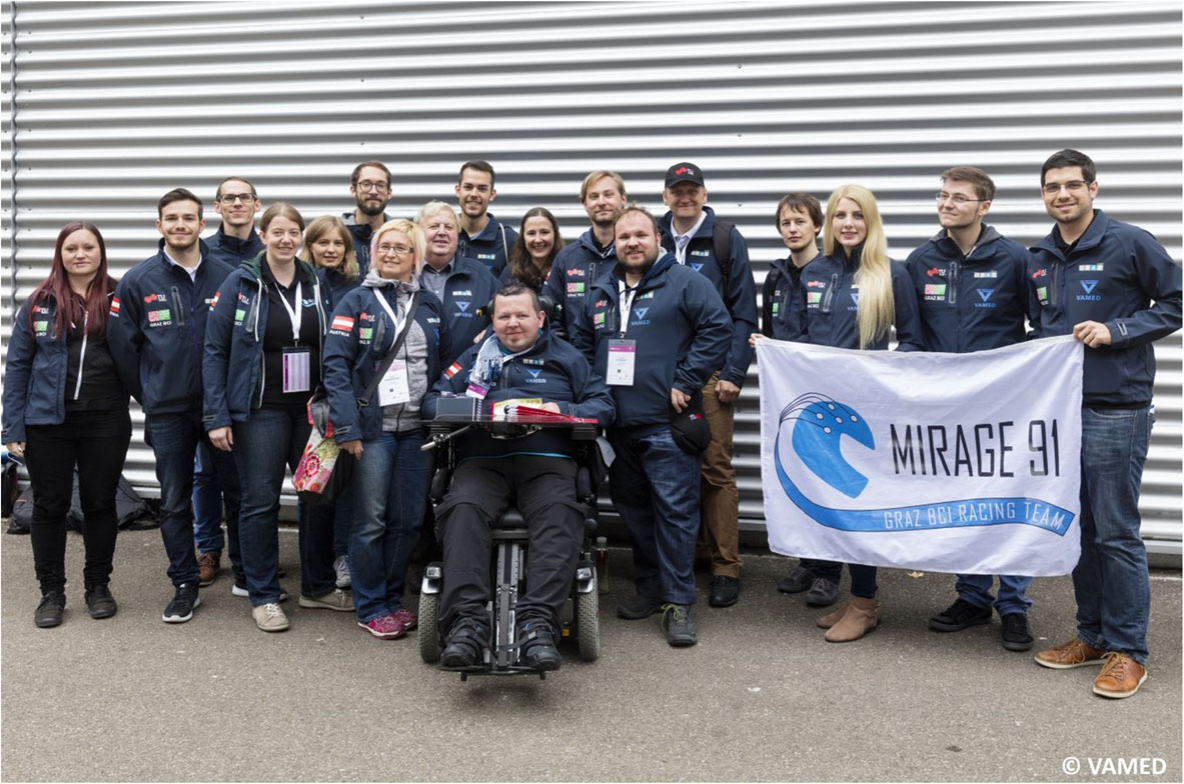
The MIRAGE91 team at the CYBATHLON 2016.

With this project, we were able to attract students to make their first experiences with BCI research, to work with pilots, and to meet other young scientists in an international setting. Fig. 1 shows a picture of the team, taken in Zurich at the CYBATHLON 2016.

### Pilot recruiting and status

After the rehearsal, our main goal was to identify a suitable pilot for our team. We were contacted by VAMED, an Austrian global provider in the healthcare sector. They were looking for an Austrian team participating in the CYBATHLON 2016 and they brought us in contact with the Neurological Center in Kapfenberg (NTK), where we established first contact with our pilot one year before the CYBATHLON 2016.

The pilot of the Graz BCI Racing Team MIRAGE91 was a 37 year old male. Before he received a stroke, he had been an active athlete. His discipline was luge racing on natural tracks. In 01/2014, he was diagnosed with an extended stroke of the brainstem and cerebellum (right side) resulting from a thrombosis of the basilar vein which lead to an incomplete locked-in syndrome. At hospital admission, the patient was almost completely paralyzed with little residual ability in the upper extremity. During treatment, the motor abilities have since increased to a point where he is able to operate an electric wheelchair using a joystick as an assistive device. Currently, though severely speech impaired, he is vigilant and fully aware of his environment.

### Training

Reliable BCI control is a complex mission, not only for pilots, but also from a technical point of view. Although there have been first attempts towards plug and play BCIs, we decided to closely tailor a BCI to our pilot manually [19]. Tailoring a BCI includes the technical perspective, but also other aspects, like customizing the set of mental tasks, and is referred to as user centered design [20–22].

**Fig. 2:**
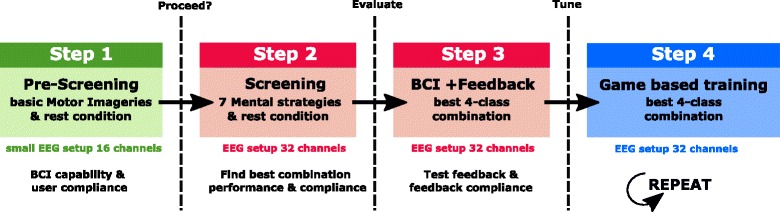
4 Stage training procedure: In pre-screening (step 1), the BCI aptitude of the pilot was evaluated. In step 2, screening, the best 4-class combination out of a pool of mental strategies was identified. Stage 3 tested the pilot’s compliance with receiving feedback. Based on all collected data, a closely tailored BCI was implemented. In stage 4 the pilot started training with the competition game

Based on findings in previous studies [23–26] as well as our own experiences, we came up with a four step plan [27] to guide our pilot towards achieving reliable multi-class BCI control (see Fig. 2).

In the first step, we started with a pre-screening session to evaluate whether the pilot candidate is able to trigger discriminable sensorimotor rhythm (SMR) based brain patterns on demand. We were also interested in the pilot’s ability to concentrate and to understand our instructions. This step was a milestone for both the pilot and the MIRAGE91 Racing Team, to decide whether continued effort and training was reasonable.

Studies from Friedrich et al. [25] and Müller-Putz et al. [23] indicate that there is a large number of mental tasks which induce changes in oscillatory EEG components. These changes can be utilized to discriminate different mental tasks. However, their findings suggest that discrimination performance varies between task combinations and individual users. As a second step in our tailoring process, we conducted a screening of eight different mental tasks for our pilot to find sets of four tasks with distinct patterns. Ultimately, the pilot chose the most comfortable 4-task combination out of the best performing sets.

In step three, we put our findings to the test in an online BCI system. For the first time, the pilot received feedback according to his mental actions. We were primarily curious about the performance of the chosen 4-task combination, but also about the pilot’s compliance to feedback.

In the fourth step, we used the information gathered in the previous steps to optimize the BCI system for our pilot, including modern machine-learning methods [23–26, 28, 29], transfer of calibration trials from one session to the next to reduce setup time, and a customized 4-task combination. This tailored setup was eventually used to perform training sessions over a period of six months.

#### Step 1: Pre-screening

It was necessary to carry out a pre-screening of the pilot candidate in order to assess his suitability for the discipline. Three points had to be clarified: (1) The pilot’s ability to understand and perform the requested tasks, (2) his capability to elicit distinguishable brain patterns and (3) the effects of the performed tasks on the pilot. It was necessary to assure that executing the tasks did not cause harmful side effects such as spasms or discomfort for the pilot. We performed two pre-screening sessions on two separate days.

**Fig. 3:**
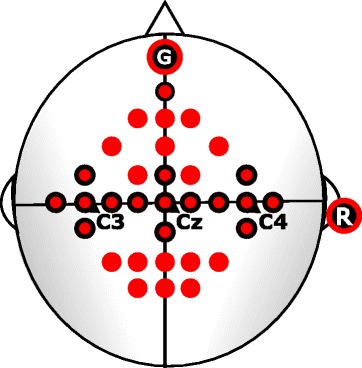
Electrode setup: The 16 black-outlined electrodes were used for the pre-screening stage. The consecutive stages used all plotted electrodes

We recorded EEG using a biosignal amplifier with 16 active electrodes (g.tec, Austria) at a sample rate of 512 Hz. A notch filter (50 Hz) was used in the recording process along with a bandpass filter with cutoff frequencies of 0.1 and 100 Hz (8th order butterworth filter). EEG was recorded at the positions C3, Cz and C4. We placed four additional electrodes in an equidistant setup (2.5 cm) orthogonally around each position to allow for Laplacian derivations. The one remaining electrode was located at position AFz. Reference and ground electrodes were placed on the right earlobe and frontally, respectively. The whole electrode setup is shown in Fig. 3.

**Fig. 4:**
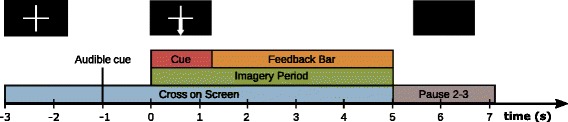
Graz-BCI Paradigm: At second −3, a cross appeared on the screen, followed by an auditory cue at second −1 to get the attention of the pilot candidate. At second 0, the cue is presented, followed by a five second imagery period. Depending on the cue, the pilot performed the designated task for the whole imagery period

In both sessions, the standard Graz-BCI paradigm with three classes was used [6] (see Fig. 4). At second −3, a cross was displayed on the screen followed by an auditory cue at second −1 to get the pilot candidate’s attention. At second 0, a visual cue was presented for 1.25 s instructing the candidate on the designated task. In the pre-screening, we chose abstract arrows as cues. The pilot candidate performed the task for the next 5 s, until the cross vanished at second 5. Thereafter, an inter-trial break of 2–3 s followed to permit the pilot candidate to move his eyes freely.

In the first session, four consecutive runs were recorded. Each run comprised 10 trials per class (TPC) in pseudo randomized order, i.e. in total, 40 TPC were performed. We focused on three different motor imagery tasks: repeated opening and closing of the (1) right and (2) left hand and (3) plantar flexion/extension of both feet. For the second session, we changed the tasks to two motor imagery classes (right hand and both feet) and one rest class. During the rest trials, the designated pilot was instructed to relax and perform no mental imagery. This time, 50 trials per class (five runs) were recorded.

We rejected artifact contaminated trials using statistical parameters: (1) amplitude threshold (amplitude exceeds +/− 100 μV), (2) abnormal joint probability and (3) abnormal kurtosis. As threshold for the latter two, we used four times the standard deviation (STD) [19, 28].

**Fig. 5:**
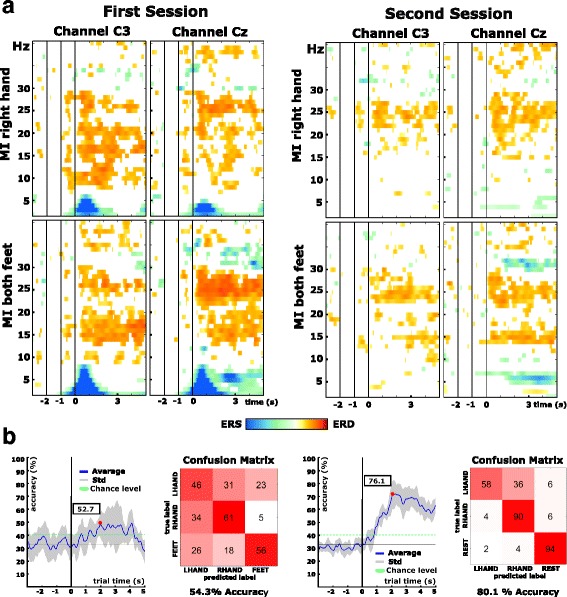
Pre-screening results for session 1 (left) and 2 (right): **a** ERD/ERS maps calculated for right hand and both feet MI (left side). **b** Cross-validation accuracy curves summarize the course of classification accuracy over the average trial (chance level calculated using an adjusted wald-interval, alpha = 0.05). The confusion matrix summarizes the performance of the classifier across a session’s trials

We calculated time-frequency maps using 5 point Laplacian derivations [30] for positions C3, Cz and C4. A bandpass filter between 2 and 40 Hz (Butterworth, causal, 6th order) was applied and data were cut into segments lasting from 3 s before until 5 s after the cue. Event-related desynchronization and synchronization (ERD/S) of the designated pilot were analyzed [31] using a reference interval of second −2 to second −1 before the cue. The results were tested for statistical significance with t-percentile bootstrapping at a significance level of alpha = 0.05. Significant differences are shown in color in Fig. 5a.

We were also interested in how well the recorded mental tasks were discriminable against each other. Therefore, the data were bandpass-filtered between 6 and 35 Hz using a 4th order zero-phase butterworth filter. To avoid overfitting, we separated trials into training and test data using 10 times 5 fold cross-validation. In each fold, we trained regularized common spatial patterns filters (CSP) [32–34] for each possible class combination using data from second 1 to 4 with respect to the visual cue. From each CSP class combination we took the first and last two projections (which hold the most discriminative information for the class combination) and calculated 12 logarithmic bandpower projections using a moving average filter over the last second (step size: 1 sample). In a second step, training of a shrinkage Linear Discriminant Analysis (sLDA) classifier [35] was performed using bandpower features 2.5 s after the visual cue. These calculated models were then applied to the (fold-specific) test data to assess fold performance. To evaluate the overall class performance, we also calculated the confusion matrix over the feedback period from second 1 to 4. A trial was marked as correct if the majority of predictions within the trial were correct. All trials were thus evaluated. We performed row-wise normalization and calculated the percentage for each matrix value.

Analysis of the recorded data showed that the pilot candidate was able to generate distinguishable brain patterns in both sessions (see Fig. 5). We had the impression that the pilot was excited and nervous during the first session, which we attributed to the novelty of the situation and his first contact with BCI technology. This perceived excitement and nervosity may be a reason for the low-frequency EOG artifacts in the time-frequency maps right after presentation of the cue (second 0). Classification accuracies exceeded chance level in both pre-screening sessions. Chance levels were calculated using an adjusted Wald interval with an alpha of 0.05 [36].

For the first session, the maximum accuracy was 52.7% approximately two seconds after cue presentation. Analysis of the confusion matrix showed that left hand motor imagery classification performance was lowest of the tested mental tasks. Since results from the first session already indicated that the pilot candidate was able to produce distinguishable patterns, we exchanged left hand motor imagery with a rest class. In the second session, the pilot candidate was more focused on the task and was able to reduce eye movements. Time-frequency analysis showed more distinct patterns and no sign of contamination due to eye movements. The performance of this new class combination (right hand, both feet, rest) exceeded results from the previous session. The maximum accuracy was 76.1%, again approximately two seconds after the visual cue. Analysis of the confusion matrix showed an increased false positive rate between right hand versus both feet, however both classes were well discriminable against the rest condition.

The designated pilot experienced no harmful side effects or discomfort and was indeed vigilant and concentrated in both sessions. In mutual agreement between the pilot candidate and the team we decided to continue training and he became the official pilot of the team.

#### Step 2: Screening

In the screening step, the most suitable class combination for our pilot had to be found. The four chosen classes should yield high classification accuracy and at the same time be comfortable for the pilot.

The electrode setup for the screening process had to be extended since non-motor tasks were now included in testing. We used 32 active electrodes by adding additional electrodes to frontal and parietal areas (see Fig. 3).

We chose seven different mental tasks, in accordance with [25], and a rest condition.

1. **MI of the right hand (HAND):** imagination of repeated squeezing of a rubber ball.
2. **MI of both feet (FEET):** imagination of repeated plantar flexion/extension of both feet.
3. **Word association (WORD):** producing a series of words starting with a letter shown on the screen.
4. **Mental subtraction (SUB):** repeated chain-like subtraction of the same number, starting with one equation presented on the screen.
5. **Auditory imagery (AUD):** imagination of singing a certain song.
6. **Spatial navigation (SPATNAV):** imagination of moving from one room to another one in one’s home.
7. **Mental rotation (ROT):** imagination of rotating a 3D object like a cube.
8. **Rest (REST):** no distinct mental action, focus on the screen, prevent eye movements

**Fig. 6:**
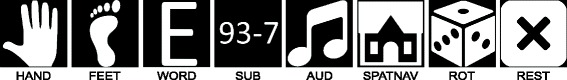
Icon set for the screening paradigm (left to right): (1) MI right hand, (2) MI both feet, (3) word association, (4) mental subtraction, (5) auditory imagery, (6) spatial navigation, (7) mental rotation, (8) rest

For each task, we recorded 45 TPC in nine consecutive runs using the Graz-BCI paradigm. All cues were presented as white symbols on the screen in pseudorandomized order (see Fig. 6). Since we wanted to find the 4-class combination with the highest performance, we conducted an analysis for each possible 4-class combination (70 in total) to determine class discriminability. Again, we bandpass-filtered the data between 6 and 35 Hz using a 4th order zero-phase Butterworth filter and a 10 times 5 fold cross-validation technique to avoid overfitting.

In each fold, we separated the trials in training and test data. We trained CSP filters on (training) trial data from one second to three seconds after the visual cue on every possible class combination. We took the first and the last two CSP projections and calculated logarithmic bandpower projections similar to the pre-screening. Thereafter, an sLDA classifier was trained using the training data on bandpower features located 2.5 s after the visual cue and evaluated on the test data of the fold. In this way, we acquired 50 fold-specific performance results from which we took the mean and the standard deviation.

**Fig. 7:**
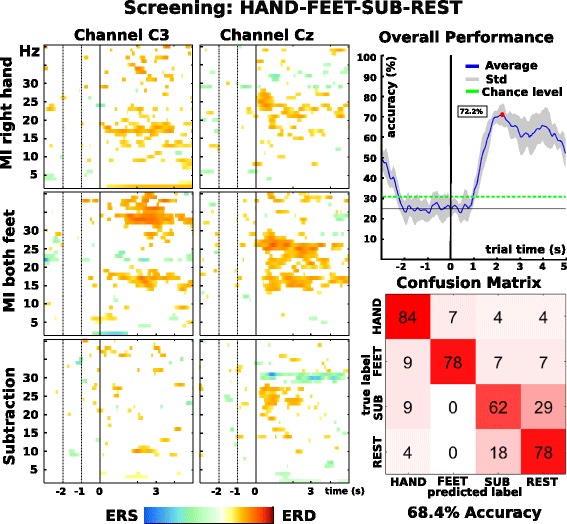
Screening results for the best performing combination Hand - Feet - Subtraction - Rest. Left: Time-frequency maps for motor imagery tasks hand, feet and subtraction. Top right: Offline calculated cross-validation accuracy curve and its peak at 72.2% (red dot) (chance level calculated using an adjusted wald-interval, alpha = 0.05). Bottom right: Confusion matrix for second 1 to 5, values in percent

Peak and median accuracies of the best five 4-task combinations are shown in Table 1. A detailed overview of the offline performance over all trials can be seen in Fig. 7. Similar to the pre-screening, we calculated a confusion matrix to assess individual class contribution to the overall performance.

**Table 1:** Peak and median accuracies (second 1 to 4) across 4-task combinations, achieved during the screening session

Combination	Peak accuracy (%)	Median (1–4) (%)
Hand-Feet-Subtraction-Rest	75.6 STD +/− 5.0	66.1 STD +/− 8.52
Hand-Feet-Word-Rest	72.2 STD +/− 11.0	63.3 STD +/− 8.92
Hand-Feet-Spatial-Rest	68.4 STD +/− 5.0	56.1 STD +/− 7.5
Hand-Feet-Rotation-Rest	68.9 STD +/− 6.0	56.1 STD +/− 6.9
Hand-Feet-Subtraction-SpatNav	67.8 STD +/− 5.0	60.0 STD +/− 8.76
Hand-Feet-Subtraction-Aud	67.2 STD +/− 7.0	59.4 STD +/− 7.5

All accuracies were estimated via 10 times 5-fold cross-validation (CV). The single accuracies of the CV were used for estimating the standard deviation of the accuracies (STD). The combination Hand-Feet-Subtraction-Rest worked best for the pilot, not only in peak accuracy, but also in median accuracy over the feedback period from second 1 to second 4

With this approach, we found a number of distinguishable 4-task combinations, the best-performing one being MI of the right hand, MI of both feet, mental subtraction and rest (HAND-FEET-SUB-REST), resulting in a peak accuracy of 75.6%, approximately two seconds after cue presentation. The calculated confusion matrix revealed high true positive rates for the motor imagery classes and the rest condition. Diminished true positive rates were observed for the class mental subtraction, which showed increased rates of false positives and false negatives in connection to the rest class, as shown in Fig. 7.

In general, the five most promising 4-task combinations were within one standard deviation with respect to the best performing combination (Table 1). Interestingly, all five best 4-task combinations out of 70 in total involved both tested motor imagery classes. This conforms with the findings of Friedrich et al. [25], where motor imagery tasks were part of the best class combination for every tested subject.

We also performed time-frequency analyses focussing on the motor areas to monitor any changes in patterns over multiple sessions. In screening, these maps again showed stable, distinguishable results and were comparable to previous sessions.

We discussed the most promising class combinations with our pilot, and agreed to proceed to the next step with the most performant one.

#### Step 3: Online BCI with feedback

With the 4-task combination and basic parameters at hand, we brought the pilot into first contact with a closed loop online BCI system. The main task was to assess the pilot’s performance in an online scenario, but also his compliance with receiving feedback.

We kept the settings for the amplifier and electrode setup similar to the screening session. The best 4-task combination of the screening (HAND-FEET-SUB-REST) was used to control the BCI. Again, we used the Graz-BCI paradigm to acquire calibration data. However, visual cues in the paradigm were now color-coded according to the four action pads of the CYBATHLON game: gray for REST, yellow for SUB, magenta for FEET and cyan for HAND. With this paradigm, 50 trials per class were recorded as calibration data. During the imagery period from second 1 to 4, we used a horizontal bar graph to present feedback. The length of the bar represented the user’s performance and was proportional to the amount of correct classifications during the previous second.

Again, as already performed in the previous steps, we applied a statistical outlier rejection to exclude trials which were corrupted by artifacts [19, 28]. It discarded approximately 10% of the trails. They were evenly distributed across conditions. We replaced the zero-phase bandpass filters of the previous steps with causal implementations to accomplish consistent characteristics between training and the online BCI. Subsequently, CSP filters were trained, using trials from second 1 to 4 and all possible class combinations for the four classes (6 combinations total). 24 logarithmic bandpower projections were calculated from the first and last two projections of every CSP filter. An sLDA classifier was trained on features extracted from 2.5, 3.5 and 4.5 s after cue presentation. Both the CSP filters and the sLDA classifier were thereafter used in an online test period of additional 40 trials per class during which the pilot could track his performance through the presented feedback. Again, we calculated the accuracy over all online trials as well as the confusion matrix as already described with respect to the pre-screening. Furthermore, we had a close look on the time-frequency maps, which were calculated in a similar manner as in pre-screening.

**Fig. 8:**
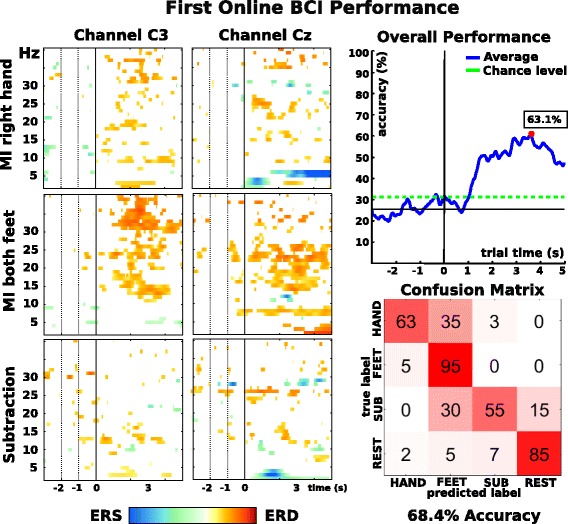
First online BCI performance Left: Time-frequency maps for motor imagery tasks hand and feet and subtraction. Top right: Online performance results peaked at 63.1% (red dot) (chance level calculated using an adjusted wald-interval, alpha = 0.05), the calculated chance level lies at 31.2%. Bottom right: Confusion matrix for second 1 to 5, values in percent

Our first attempt at an online BCI incorporating the designated 4-task combination showed promising results (see Fig. 8). Performance peaked at 63.1% for the online feedback period and at 68.4% in trial based evaluation, which exceeded the calculated chance level of 31.2% (alpha = 0.05, adjusted Wald interval). The peak accuracy maximum was postponed by almost 1.5 s compared to the offline analysis. We hypothesize that the different features (3 time points in online scenario vs. 1 time point in offline analysis) caused that change. Analysis of the confusion matrix shows high true positive rates for classes FEET and REST, while false positives primarily occurred for the combination HAND versus FEET, and SUB versus FEET.

Comparing offline calculated results (see Step 2, Screening) and online performance actively achieved by the pilot, we encountered a notable performance drop. Changes in the pilot’s task involvement (due to feedback) could have altered the feature distribution and therefore lead to an suboptimal decision boundary of the trained classifier. This effect has already been discussed by Samek et al. [37] and reconfirmed our own experiences with this delicate transition.

Inspection of the time-frequency maps of the motor task again showed stable patterns in beta/high beta range for class FEET, which is consistent with observations in previous steps (see Fig. 7, Screening). For class HAND, we could also observe similar - though less pronounced - patterns as in screening.

The pilot, who received feedback for the first time, enjoyed the process and was compliant to continuing his training with feedback. During measurements, he was concentrated and tried to avoid artifacts such as eye blinks or swallowing.

#### Step 4: BCI game

After the feedback session, the training was changed to include the actual CYBATHLON game. Since in the Graz-BCI paradigm, feedback was abstract and simplified, we wanted to assess possible distractions for our pilot under game conditions, and the pilot should get used to the game as soon as possible. However, the main goal of this step was to train the game situation for the competition.

Each BCI game session consisted of two consecutive steps. The first step was without feedback (the game avatar was not controlled), to collect data for BCI calibration. The pilot was instructed to start mental imagery as soon as his avatar reached a new action pad until it passed half of it. Thereafter, he should relax until the next action pad. The game sent triggers via UDP to mark the start of a new action pad, which we used to segment the recorded EEG data. In the second step, the pilot used the BCI to control the avatar in the BCI game - this step was the actual competition training.

**Fig. 9:**
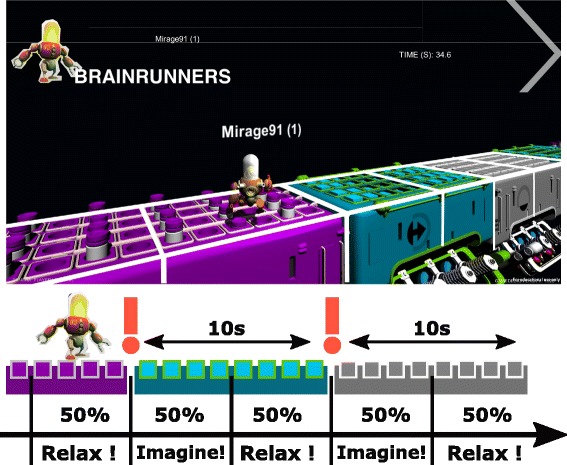
BrainRunners training paradigm: For data collection, the pilot was instructed to perform the mental task on the first half of the action pad and relax on the second half. In this manner, the pilot performed 5 s of the indicated mental task (pad color) and had a break of 5 s until the next mental task

However, it was very unpleasant for our pilot to redo the complete collection of calibration data every training session - one run encompassed 10 TPC à 10 s, i.e. a complete training session amounted to approximately 35 min, excluding breaks between runs. To shorten the recurrent calibration time in the following sessions, we decided to include 30 TPC from the respective previous training sessions and to record only 30 new TPC for calibration in each session. Hence, in each session the number of calibration trials was 60 TPC, 30 from the respective previous session and 30 TPC of the current session. This protocol reduced the calibration time by 40%. To counteract session to session transfer effects, we decided to normalize EEG channels according to the variance of a resting period recorded at the beginning of each training session. However, this normalization step was included based on theoretical considerations only, and was not evaluated regarding its influence on, for example, classification accuracy. Figure 9 shows the paradigm for training with the game, where imagination and relaxation periods alternate on every action pad.

**Fig. 10:**
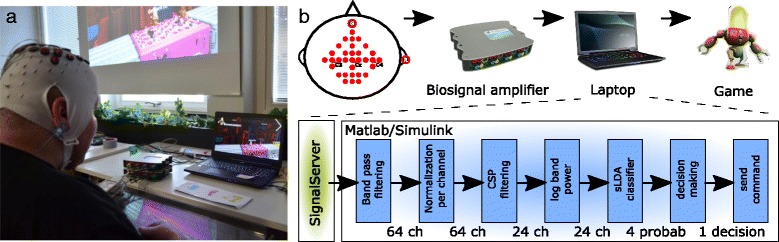
**a**. Pilot using the MIRAGE91 BCI. **b**. Schematic of the MIRAGE91 BCI

The BCI system for the game introduced new signal processing steps (see Fig. 10): First, EEG data were bandpass-filtered in two bands, between 8 and 16 Hz and 16–30 Hz, to separate the alpha and beta bands. Then, we normalized the filtered channel signals by their respective resting variance to reduce the influence of high variance channels. After that, we performed spatial filtering with shrinkage regularized Common Spatial Patterns (sCSP) in a one class vs. one class manner, separately for both frequency bands [34]. Four spatial filters, the filters corresponding to the two largest and the two smallest eigenvalues, were used per CSP model, leading to 48 features (6 class combinations × 4 filters × 2 bands). Then, we calculated the logarithmic bandpower over one-second sliding windows and used an sLDA classifier to calculate class probabilities [35]. If the one-second averaged class probability of any of the four classes exceeded a class-specific threshold, the corresponding command was sent to the game. Five times five fold cross-validation on the training data was used to estimate the mean and variance of the class probabilities, and therefore a potential bias of the classifier towards specific classes. The class-specific thresholds were set manually by a technician to counteract bias. One to two BCI game runs, played immediately after training, were the basis for further manual adjustment.

To meet CYBATHLON requirements, we added a real-time artifact detection system. It relied on two features. The first feature was an eye blink detector. Blinks were detected by comparing the power of bandpass-filtered (1–10 Hz) activity at electrode AFza to a threshold. If the threshold, equaling three standard deviations above the mean resting EEG bandpower, was exceeded, the decision making output was blocked. The second feature was checking for deviations of the ongoing EEG. Similar to [38], we modeled the EEG as an autoregressive (AR) process of order 10. The resting EEG was used to fit AR process coefficients for each EEG channel separately. The corresponding inverse finite impulse response (FIR) filters were applied to the ongoing EEG to linearly predict the next sample. If the prediction error exceeded three times its standard deviation, which was estimated using the resting EEG, the decision making output was blocked. The inverse filter and the threshold were adaptively adjusted throughout the session to compensate for slow changes in the statistical properties of the EEG.

In the upcoming months, we conducted regular training sessions with our pilot using the game. After a couple of training sessions, the pilot expressed doubts regarding our choice of the 4-task combination. In particular, he became more and more unhappy with the rest condition. The lack of focus (“thinking of nothing”) on a task did not fit his personal preferences. Therefore, we decided to replace the rest class with auditory imagery. In our screening session, the combination HAND-FEET-SUB-AUD was among the best performing combinations (see Table 1).

**Fig. 11:**
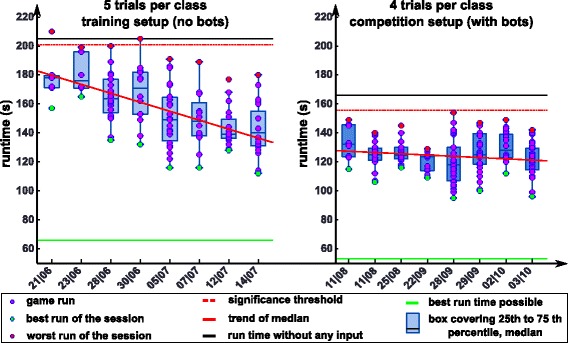
Training session results of the last months before the competition. The left plot shows training session dates on the x-axis and game runtimes (in seconds) on the ordinate for game runs with 5 TPC. The significance threshold (red dotted line) is the median runtime that the BCI system achieved with a random input signal. The right plot shows the results in later trainings, after we changed to 4 TPC to be compliant with the settings during the competition. This also meant that we added bots to familiarize the pilot with the situation in the arena. Game results of every session are summarized using boxplots. The best and worst run of a session is displayed in green and red, respectively

The training progress over the last four months before the championship is summarized in Fig. 11. It illustrates the evolution of runtimes across recording sessions. The runtime is defined as the time it takes the avatar to cover the distance between start and end of the track. The median runtime per session declined steadily, indicating that the pilot’s skill improved over time. During training, he was concentrated and compliant to our instructions. The number of games played varied according to the pilot’s motivation on the respective day. At first, we scheduled a training session twice a month, and as the CYBATHLON competition came closer, we increased the training frequency.

Analyses of the game runtimes of our pilot showed a significant linear trend (*p* = 0.00017) of the median towards faster runtimes for training sessions until end of July, Fig. 11 (left). In August we started training with the final game, including computer-controlled competitors and 4 TPC instead of 5. Right up until the competition, the pilot was able to maintain his median performance, with a non-significant trend towards better runtimes (*p* = 0.12772), Fig. 11 (right).

## Cybathlon

**Fig. 12:**
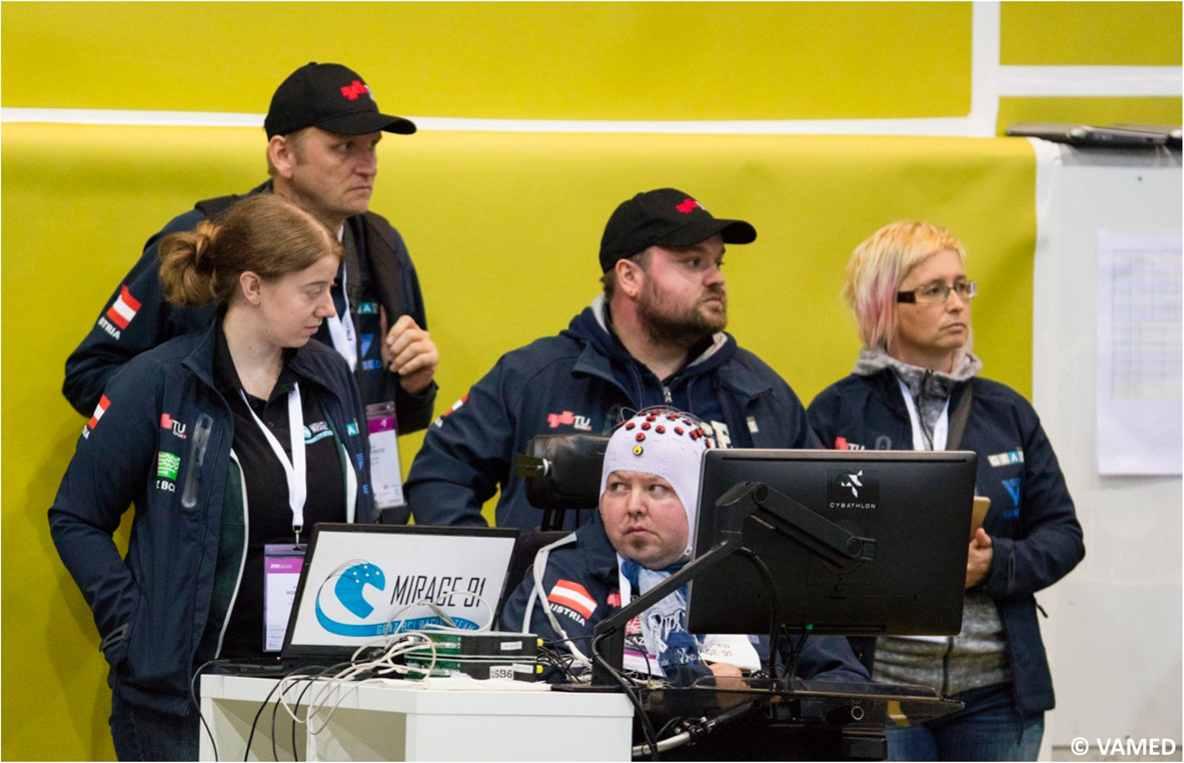
Pilot and team members in the arena minutes before the BCI Race. The teams were provided with a monitor, which could be placed in front ot the pilot, a shelf to place their equipment, a power strip and a network cable

Due to a compulsory medical check, our pilot had to be in Zurich one day before the CYBATHLON 2016. The organizers provided a container next to the stadium for each of the 11 BCI Race teams, intended as a reduced noise environment for focused pilot preparation and BCI calibration before the BCI Race. Since the teams were also invited to use this container the day before, we used the opportunity to do a training session and recorded four runs in the container. On the competition day, two hours before the BCI Race, two team members and the pilot started with the preparation for the race. The cap was mounted and the final three training runs were recorded. Since our system processes data from multiple sessions, we added the last three training runs from the previous day. After system calibration, our pilot played the game five times and was able to achieve runtimes of around 120 s. They matched with the performance of the previous sessions (see Fig. 11). The pilot and the supporting team members were then asked to proceed to the arena (Fig. 12). There, they connected the BCI system to the official game. In this warm-up phase, lasting 30 min, the pilot could already send commands to the game and receive visual feedback. We used this time to assess system functionality. The pilot was able to trigger specific commands a supporting member asked him to think of. We were ready for the countdown to the race - the race we all were working towards for the last two years. Soon after its start, we realized that during this utmost important game our system elicited novel disadvantageous behavior. Its output was strongly biased towards a single class, resulting in a runtime of 196 s. However, the qualification times for the finals were in the interval (90, 165) seconds. As a consequence, we failed to qualify for the final races and finished on 11th place. More information about game results are available online at the official CYBATHLON website [39].

**Fig. 13:**
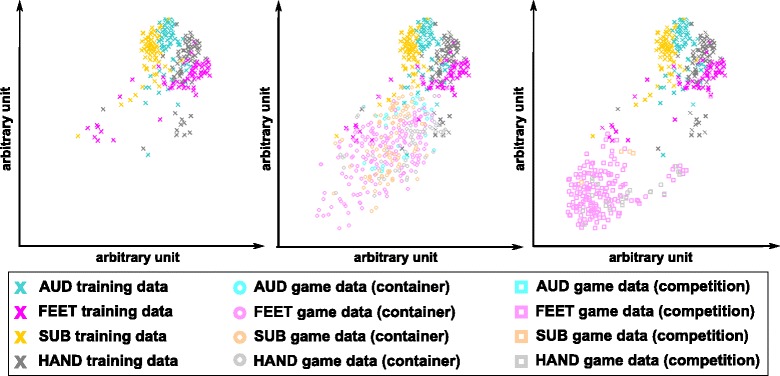
2D representation of the 48-dimensional feature space computed with t-SNE: Each point corresponds to an observation. The smaller the distance between the 2D points, the closer they are in the 48D feature space. (left) Data of the six calibration runs (crosses) on which the sLDA classifier was trained on. The training labels were used to color-code the 2D points. The other plots additionally summarize observations during games in the container (middle, circles) and the arena (right, squares). We used the sLDA classifier output to color-code the points for this data

A first analysis of the recorded signals revealed that the strong bias towards the class FEET started 3 min before the final game. However, a check of the raw EEG signals and their power spectrum did not reveal differences between the games played in the container and the game in the arena. A more detailed analysis lead to the conclusion that the feature distribution had changed considerably between training and the games. Figure 13 depicts the difference. It shows 2D representations of the 48-dimensional feature space. We applied t-Distributed Stochastic Neighbor Embedding (t-SNE) [40], an unsupervised nonlinear dimensionality reduction technique, to compute the projection. In t-SNE, the high dimensional data are represented by 2D points such that similar data points are modeled by nearby 2D points. The leftmost plot summarizes the training data distribution. The labels were used to color-code the 2D projections, indicating that the training data contained discriminative information. The plot in the center adds data obtained during the five games in the container (circles). Their distribution center is shifted compared to the training data. Due to a lack of true labels, we used the sLDA classifier output to define a point’s color. It shows that the classifier selected class FEET (magenta) for half the data points, which indicates that the bias started in this stage. During the game in the arena (rightmost plot), the distribution shifted even further away from the training data. As a consequence, FEET was triggered 85% of the time, which in turn resulted in poor game performance.

### Error analysis and potential improvements

We identified several possible sources that could partially explain the changes of the feature distribution. The non-stationary nature of the EEG might have resulted in a variation of baseline activity in the frequency bands of interest [41]. This is unlikely to explain the drastic shift between training and container game data, since these games were played immediately after training and the variation in distribution between the individual games played in the container was negligible. However, we cannot rule out a significant effect on the arena game due to the 45 min gap in between. We also suspect a contribution of the transfer from the open loop system for gathering calibration data to the closed loop system when playing the game. Other studies reported similar shifts between calibration and feedback runs [37, 42]. Lastly, the novel situation in the arena and a possibly different arousal level could have had a negative effect as well. We therefore investigated the heart rate of our pilot during the event as an indicator for the arousal level. A prominent Electrocardiography (ECG) artifact in several electrode signals allowed us to perform this analysis. The pilot had a heart rate of around 97 beats per minute (bpm), during training and playing the BCI game in the container. During the warmup in the arena (~45 min), his heart rate was initially at a similar level. Three minutes before the qualification game, it started to increase peaking at 132 bpm at race onset and returning to 100 bpm at its end. We did not observe this phenomenon in the last training sessions back in Austria, where he had a heart rate of approximately 95 bpm during training and playing the game. Due to the novel situation in the arena surrounded by hundreds of spectators and the increase of heart rate, it is plausible that our pilot got nervous. The phenomenon can only explain the performance drop to a limited extent, since the bias of the classifier already started in the container.

Our experiences in the event showed that the transition from system calibration to playing the game is critical. Unfortunately, we did not simulate long breaks in between the two stages in our training protocol. This fact will be considered in the design and evaluation of the next BCI system. Our findings also indicate that the difference between our custom training paradigm (without feedback) used for calibration and the actual game might be disadvantageous since the dynamics differ substantially [43]. A co-adaptive training paradigm, implemented in the game environment, could help to mitigate the transition effects [28, 44, 45]. An adaptive system could additionally track slowly varying changes in the ongoing EEG that result in a shifted feature distribution [42]. However, robustness to outliers is a critical requirement for the optimization algorithm.

The limited robustness of state of the art BCI systems to new environments/situations is in general a major impeding factor to move BCI technology out of the lab to the real world [46]. Robustness in the sense of the pilot’s performance fluctuations (e.g. within session variance in Fig. 11) can be related to the phenomenon of intra-subject performance variation [47, 48]. Intra-subject BCI performance has been reported to correlate positively with psychological states such as motivation [49]. It is reasonable to assume that the psychological states relate to physiological changes. Indeed, [50] identified that frontal gamma activity, which is related to attention, plays a critical role in motor imagery function. Monitoring these physiological state changes during BCI operation could improve inference of the pilot’s state and in turn allow us to provide immediate feedback why BCI performance might have declined or increased. This information has the potential to facilitate the pilot’s learning process, and consequently reduce the variance of the results reported in Fig. 11.

Moreover, we believe that extending the pilot training by including sessions in a setting resembling the competition environment, i.e. races with human competitors and a sizeable audience, may help the pilot himsef to better prepare for the race mentally. Such training sessions could give him the opportunity to practice ignoring the noise and excitement around him, and to retain a calm and focused mindset, in order to produce reliable imagery patterns.

## Discussion

### Organizational challenges

Organizing pilot training in our own facilities on regular basis was out of the question since the pilot lived a good two hours drive away from our lab. The Neurological Center in Kapfenberg (NTK) became the pivotal location between the team and the pilot since it was located midway between the pilot’s home and the team, and the pilot was receiving rehabilitation therapies there on regular basis. With support by the chief of medicine, we managed to establish regular BCI training in its facilities.

We started with initial training sessions twice per month, during which three team members worked with the pilot. Training lasted approximately three hours per session, plus two hours of driving. In the last months before the CYBATHLON, the training schedule was intensified to a frequency of at least one session per week, twice when possible. In the week before the CYBATHLON, we organized a four day training camp in the pilot’s hometown where we trained twice a day. All in all, training was indeed time-consuming and demanding to both the team and the pilot. A good compensational factor was our large team. Summed up, 15 people actively participated in all aspects of preparation. Therefore, we were able to form small subteams to alternatingly conduct training sessions. This helped in consolidating curricular studies and the engagement for the team.

Organizing the trip and accommodation for both the pilot and the team was another organizational challenge. While taking part in the CYBATHLON rehearsal had prepared us for a lot of the organizational aspects of the event, our pilot’s travel and accommodation required additional arrangements. Evidently, he needed a hotel close to the competition arena with handicapped accessible facilities.

Our pilot was accompanied by two caregivers, his wife and his father, as well as ample amounts of equipment for his mobility and care, e.g. two different wheelchairs and assistive devices for daily hygiene. Together they traveled by car, including an additional trailer for the pilot’s equipment. The pilot and his caregivers arrived two days before the event to conduct the obligatory medical check and prepare for the race.

The majority of the MIRAGE91 Racing Team traveled to Zurich on the day before the competition, while some team members went there two days earlier to prepare for the race with the pilot and to attend the CYBATHLON Symposium.

### Public awareness

The communication of visions, ideas and results of science is one of the major challenges every scientist is faced with. While writing scientific papers is part of the core daily work of a researcher, reaching the general public works on a different level of communication, which is less detail-oriented and has a higher emphasis on entertainment value.

The CYBATHLON creates a unique opportunity to present new technology in action, while at the same time actively involving potential end users of the technology. It raises awareness for the daily life challenges of pilots and fuels interest in the advancement of research. Moreover, collaborating with a pilot over an extended period of time gives teams valuable insights into their needs and their reality of life. On the other hand, it offers an opportunity to the pilots themselves to use e.g. a BCI first-hand and get into direct contact with research aimed at making their lives better.

Both our preparatory phase and the competition itself were accompanied by a fair amount of national media coverage. Austrian television as well as several Austrian newspapers reported on our preparations with our pilot in the months leading up to the competition, and many outlets followed up with reports from the event, including Austrian radio, who interviewed team members at the venue. Furthermore, our sponsor VAMED produced a video promoting the CYBATHLON, as well as our pilot and team.

Furthermore, the competition received full day live TV coverage via 3Sat (broadcasted in Austria, Germany, Switzerland) and Swiss television, and in the form of video clips from BBC. Media from all over the world were highly interested in the event. A variety of different online and print media released special reports and articles about the CYBATHLON 2016 and participating teams [32].

### Future

The immediate goal of the MIRAGE91 team was the participation in the CYBATHLON 2016 BCI Race with a student team and a handicapped pilot. Following the CYBATHLON 2016, the team is facing the challenge of changes in the team. As is the nature of a student team, people will leave and new members will join the team. With the prospect of the next full-scale CYBATHLON 2020, and potentially a CYBATHLON BCI Series 2019 in Graz, we are going to continue with the MIRAGE91 Team and make an effort to attract new team members as well as pilots.

Starting ahead to these new challenges, we will review our CYBATHLON 2016 system and make concrete plans on how to improve the system in terms of signal processing, stability, artifacts, but also on new ways of pilot involvement and pilot training.

## Conclusion

In this work, we shared our experiences made at the CYBATHLON 2016. We showed our efforts, starting from forming the team, via our multi-stage approach for tailoring a BCI to the pilot, up to the participation in the CYBATHLON 2016 itself, with its organizational challenges.

One of the most important things we found was that no matter how well the system and the pilot perform beforehand, there is a considerable measure of uncertainty in the performance at such an event. Even though our race performance was below our expectations, participating in the first CYBATHLON was a great experience for all of us. We learned how to work in a team towards a common goal and how to organize things together with our pilot. We were able to turn our theoretical knowledge into practice, try out new things and become familiar with the field of brain-computer interfaces in an interactive and hands-on manner. Numerous smaller challenges arose during the competition, prompting us to find fast solutions and adapt to new situations. Looking back, our greatest success was to actually compete in the CYBATHLON with a motivated pilot and a working system.

## Abbreviations

AR: autoregressive
AUT: imagination of singing a certain song
BCI: Brain-computer interface
CSP: common spatial patterns
CV: cross validation
ECG: Electrocardiography
EEG: electroencephalography
EOG: Electrooculography
ERD/S: Event-related desynchronization/synchronization
FEET: imagination of repeated plantar flexion/extension of both feet
FIR: finite impulse response
HAND: imagination of repeated squeezing of a rubber ball
MI: motor imagery
NTK: Neurological Center in Kapfenberg
REST: no distinct mental action, focus on the screen, prevent eye movements
ROT: imagination of rotating a 3D object like a cube
sCSP: shrinkage common spatial patterns
sLDA: shrinkage Linear Discriminant Analysis
SMR: sensorimotor rhythms
SPATNAV: imagination of moving from one room to another one in one’s home
STD: standard deviation
SUB: repeated chain-like subtraction of the same number, started by one equation presented on the screen
TPC: trials per class
t-SNE: t-Distributed Stochastic Neighbor Embedding
WORD: producing a series of words starting with a character shown on the screen
